# Integration and functional performance of a decellularised porcine superflexor tendon graft in an ovine model of anterior cruciate ligament reconstruction

**DOI:** 10.1016/j.biomaterials.2021.121204

**Published:** 2021-12

**Authors:** Jennifer Helen Edwards, Gemma Louise Jones, Anthony Herbert, John Fisher, Eileen Ingham

**Affiliations:** a Institute of Medical and Biological Engineering, School of Biomedical Sciences, University of Leeds, Leeds, LS2 9JT, UK; b Institute of Medical and Biological Engineering, School of Mechanical Engineering, University of Leeds, Leeds, LS2 9JT, UK

**Keywords:** Decellularised tendon, Anterior cruciate ligament reconstruction, Immune response, Tissue biomechanics, Cell infiltration, In vivo biocompatibility

## Abstract

The objective was to evaluate the performance of decellularised porcine superflexor tendon (pSFT) as an anterior cruciate ligament (ACL) reconstruction device. The ACL of adult sheep was reconstructed with decellularised pSFT or ovine allograft SFT and animals sacrificed at 4, 12 and 26 weeks (n = 4 per group) for biological evaluation and 26 weeks (n = 6) for biomechanical evaluation of the grafts. Both grafts showed good *in vivo* performance with no major differences at macroscopic evaluation post euthanasia. Histopathology revealed an inflammatory reaction to both grafts at 4 weeks, which reduced by 26 weeks. There was advanced cellular ingrowth from 12 weeks, ligamentisation of intra-articular grafts, ossification and formation of Sharpey's fibers at the graft/bone junctions. Immunohistochemistry showed that at 4 and 12 weeks, the host response was dominated by CD163+ M2 macrophages and a cell infiltrate comprising α-SMA + myofibroblasts, CD34^+^ and CD271+ progenitor cells. At 26 weeks the biomechanical properties of decellularised pSFT and oSFT grafts were comparable, with all grafts failing in the intra-articular region. This study provides new insight into constructive remodelling of tendons used for ACL replacement and evidence of integration and functional performance of a decellularised xenogeneic tendon with potential as an alternative for ACL reconstruction.

## 1 Introduction

Anterior cruciate ligament (ACL) tears are a common knee injury, particularly in the young and active population. Severe tears require replacement of the ligament to restore stability, with approximately 7000 reconstructions per year in the UK [1] and over 100,000 in North America [2]. The aim of ACL surgery is to restore stability, enabling return to sport and activity, and to reduce the subsequent risk of arthritis. ACL reconstruction options include bone-patella tendon-bone or hamstring tendon autografts and allografts [3], all of which have limitations. The use of autograft tissue requires secondary surgery for the patient and carries the risk of morbidity at the donor site [4]. There is also the risk of an autograft having inadequate graft dimensions following harvest [5]. Allograft tissue avoids the problems of donor site morbidity and may allow ‘easier’ initial recovery, however the tissue remodelling process is slower compared to autografts [6] and inferior outcomes have been reported in young, highly active patients [6]. Allografts derived from older donors have lower tensile strength and load to failure, hence the use of grafts from younger donors is preferable, especially for younger patients [6], and availability of suitable allografts may be a limitation [7]. Prosthetic ligaments are no longer recommended for the reconstruction of the ACL, having a high rate of failure due to chronic inflammation and poor mechanical outcomes [8].

In response to these limitations, tissue-engineering approaches to ACL reconstruction are under investigation. Silk scaffolds formed through a variety of manufacturing methods have demonstrated promising results for ligament and ligament-bone healing in rabbit models of ACL repair [9,10]. Silk fibre scaffolds have also shown promising biological integration in a sheep model of ACL repair, although superior results were obtained in animals undergoing the addition of autologous stromal-vascular fraction to the scaffolds during implantation [11,12]. Although these studies are promising, more information is required on the biomechanical performance of these grafts in large animal models to understand their full potential in ACL repair. Despite extensive pre-clinical research into methods of augmenting ACL reconstruction or biomimetic scaffolds for ACL replacement using stem cells or platelet rich plasma, these approaches have yet to reach the clinic [7]. The ideal solution would be regenerative, “off the shelf”, mechanically functional ACL reconstruction devices in a range of graft sizes matched to patient requirements. One promising approach to creating such regenerative devices for ACL reconstruction is to utilise decellularised xenogeneic ligamentous or tendon tissue.

The process of decellularisation aims to remove the immunogenic cellular material from an animal or human donor tissue whilst maintaining the extracellular matrix native ultrastructure, composition and biomechanical properties to create a biological scaffold. Upon implantation, appropriately manufactured biological scaffolds can lead to constructive functional tissue remodelling and clinical success [13].

A range of biological scaffolds derived from human donor and animal tissues using different decellularisation processes are used extensively in surgical applications [13,14]. These are mainly derived from thin tissues such as human (eg Alloderm®, GraftJacket®) or bovine (eg TissueMend®) dermis, pericardium (eg. Veritas®) or porcine small intestinal submucosa (SIS, Surgisis®). Biological scaffolds have been evaluated for Achilles tendon reconstruction in mice (SIS [15]), rabbits (decellularised bovine tendon sheets [16]) and dogs (SIS [17]), and augmentation of rotator cuff repair in rats (decellularised human dermis [18]) and primates (decellularised porcine dermis [19]), with some evidence of constructive tissue remodelling. However, evidence suggests that tissue specific biological scaffolds which provide appropriate mechanical properties and induce appropriate cellular responses and constructive remodelling would be advantageous [14]. To date, there have been no reports of long term pre-clinical studies of homologous biological scaffolds for functional ACL replacement.

We have developed decellularisation processes using low concentration detergent (0.1% (w/v) sodium dodecyl sulphate; SDS) and proteinase inhibitors for the production of tissue specific biological scaffolds which preserve the biomechanical and biological tissue properties. Processes have been developed for dermis [20], cardiovascular [21,22] and musculoskeletal [23] applications with evidence of successful clinical translation [24,25]. The process has been adapted for a porcine superflexor tendon (pSFT) graft for ACL reconstruction [26]. This tendon has been shown to be mechanically superior to the native human ACL, even when irradiation is used as a terminal sterilisation method [27,28]. Decellularised pSFT grafts are typically 10–12 mm in diameter and demonstrate ultimate tensile strength (UTS) of 39.96 ± 3.68 MPa after sterilisation with 30 kGy gamma irradiation and 12 months storage [27]. For the human ACL, UTS has been reported between 24.4 and 37.8 MPa [29,30], suggesting that these grafts can provide the appropriate mechanical properties for reconstruction. Moreover, the grafts can be stratified by size to provide varying mechanical properties [31]. There has been one study of the performance of pSFT grafts, reportedly decellularised using our approach, in sheep over 12 weeks [32] which showed evidence of graft regeneration and functional recovery, but with limited characterisation of the cellular response. The work has demonstrated the potential of the decellularised pSFT as an ‘off the shelf’ alternative to autograft or allograft tissues for ACL reconstruction.

The primary objective of this study was to evaluate the biological integration of the decellularised pSFT as an ACL reconstruction device in the sheep knee over a six month period and to determine the biomechanical performance after 6 months implantation Secondary objectives were to (1) determine the cellular response to the implanted xenogeneic scaffold and determine whether the decellularised pSFT would be populated with endogenous cells over time using histology and a panel of antibodies to characterise the cell populations and (2) to compare the performance of the decellularised pSFT to a cellular ovine superflexor tendon allograft (oSFT). The sheep was chosen as the large animal species for this study because functional implantations of ACL replacements in sheep have been widely reported in the literature [[33], [34], [35], [36], [37]] and are recommended by international regulatory organisations for evaluation of ACL reconstruction devices [38]. Although smaller in size, the skeletally mature sheep knee is similar to the human knee in terms of anatomy, allowing anatomic graft fixation methods if smaller grafts are used [39].

## 2 Materials and methods

Unless otherwise stated, all reagents used during the study were obtained from Sigma-Aldrich (Dorset, UK), VWR International (Lutterworth, UK) or Fisher Scientific (Leicestershire, UK).

### 2.1 Procurement of porcine and ovine superflexor tendons

The hind legs of female 70 kg, four month old, Large White pigs were obtained from an abattoir (J. Penny, Leeds) within 24 h of slaughter. The superflexor tendon (SFT) is located in the porcine foot running from the toe to the ankle. The porcine SFT (pSFT) was identified, isolated and cleaned of connective tissue following removal of the skin and subcutaneous tissue. Isolated SFTs were stored at −80 °C on filter paper moistened with phosphate buffered saline (PBS; Oxoid) until required.

The hindquarters of 1–2 year old female Texel sheep were obtained from an abattoir (M&C Meats, Crossgates, Leeds) within 6 h of slaughter. Ankle skin was cleaned and removed, and the subcutaneous tissue cleaned with Betadine® before aseptic dissection of the superflexor tendons. Ovine superflexor tendons (oSFT) were stored at −80 °C.

### 2.2 Decellularisation of pSFT

Decellularisation of pSFT was carried out using a modified version of Jones et al. [26] using aseptic technique throughout. pSFT were thawed and split down the centre and the tendon halves were decellularised together to provide one sample for quality assurance (QA) and one for implantation. The tendons were split because it was deemed necessary following initial implantation trials, which revealed that a graft diameter of 6–7 mm was required for the ovine knee. Tendons underwent a further dry freeze thaw, followed by two freeze/thaw steps in hypotonic buffer with aprotinin (10 mM tris, 2.7 mM EDTA, 10 KIU. ml^−1^ aprotinin (Nordic Pharma), pH 8). Tendon halves were separated and washed 3 times in acetone (50 ml) for 1 h each at 42 °C on an orbital shaker (240 rpm; Grant, model PSU-10i) and then washed 5 times for 5 min with PBS supplemented with 10 KIU. ml^−1^ aprotinin (PBS + A). The two paired tendon halves were placed in a 150 ml pot and sonicated for 15 min in 100 ml PBS + A in an ultrasonicating water bath (VWR, model T20235X). The remaining treatments are detailed in Table 1; unless otherwise stated all steps were carried out with 100 ml of solution on an orbital shaker (240 rpm) and 10 min of sonication prior to any solution changes.

**Table 1:** Decellularisation process for porcine superflexor tendons following initial freeze/thaw cycles.

Step		Duration(s)	Details
1	Antibiotic wash solution (PBS containing 0.05 mg ml^−1^ vancomycin, 0.5 mg ml^−1^ gentamicin and 0.2 mg ml^−1^ Polymixin B)	1 h	37 °C, agitation at 80 rpm, no sonication
2	Hypotonic buffer (10 mM tris, 2.7 mM EDTA) pH 8.0 with aprotinin (10 KIU.ml^−1^)	66–72 h	4 °C
3	Hypotonic buffer with SDS (10 mM tris, 2.7 mM EDTA, 0.1% (w/v) SDS) pH 8.0 and aprotinin (10 KIU.ml^−1^)	22–24 h; two washes	42 °C
4	Hypotonic buffer, as Step 2	22–24 h; two washes	4 °C then 42 °C
5	Hypotonic buffer with SDS (0.1% w/v), as Step 3	66–72 h	42 °C
6	Hypotonic buffer, as Step 2	22–24 h; two washes	4 °C then 42 °C
7	Hypotonic buffer with SDS (0.1% w/v), as Step 3	22–24 h; two washes	42 °C
8	PBS (Oxoid) supplemented with 10 KIU. ml^−1^ aprotinin (PBS + A)	6–8 h 66–72 h 6–8 h 16–18 h	42 °C
9	Nuclease treatment (50 mM tris, 1 mM MgCl_2_·6H_2_0, 10 U.ml-1 Benzonase, pH 7.6)	3 h 3 h 16–18 h	37 °C, agitation at 80 rpm, no sonication
10	PBS	22–24 h	42 °C
11	Hypertonic (50 mM tris, 1.5 M sodium chloride, pH 7.6)	22–24 h	42 °C
12	PBS	66–72 h	42 °C
13	Peracetic acid (PAA; 0.1% v/v peracetic acid in PBS, pH 6)	3 h	27 °C
14	PBS	30 min; 3 washes	42 °C, no sonication
15	PBS	22–24 h; 4 washes	Alternated 42 °C and 4 °C
16	PBS	66–72 h	42 °C
17	PBS	22–24 h; 4 washes	Alternated 42 °C and 4 °C
18	PBS	66–72 h	42 °C

Following the final PBS wash, tendons halves were separated and packaged aseptically in custom made foil/Tyvek pouches (Riverside Medical Packaging Ltd) and stored flat at −80 °C until required for QA or shipping to NAMSA, Chass-sur-Rhone, France on dry ice for implantation in sheep.

### 2.3 Quality assurance (QA) of tissues

Decellularised split pSFT were assessed for sterility, removal of cellular material, maintenance of histological structure, total DNA content, biocompatibility and biomechanical properties.

Sterility of oSFT was tested immediately following extraction using a small piece of tissue from both the toe and ankle regions. For decellularised split pSFT, one half of the tendon was designated for QA and the other for potential use in the animal study. Sterility of pSFT was assessed following decellularisation and prior to packaging for every tendon produced. This assessment was carried out by placing a small sample of the toe region of each QA tendon in 10 ml sterile nutrient broth (Oxoid), incubated at 37 °C for 48 h and checked for signs of cloudiness or film formation. Only tendons where the QA sample showed no signs of microbial growth were used in the animal study. For all other quality assurance analyses, tissue was taken from a set of 6 QA tendon halves, which were chosen at random from the packaged tissues. As the decellularisation process had been previously established [26], it was deemed sufficient to perform checks to confirm decellularisation on a random subset of the tendons.

### 2.4 Histological analysis

Samples of decellularised pSFT from the ankle, middle and toe regions of each of the 6 QA tendon halves were fixed in 10% (v/v) neutral buffered formalin (NBF) for 96 h, prior to paraffin wax-embedding using standard techniques. Serial sections (10 μm) were cut longitudinally with sections captured for staining every 200 μm for 1 mm. Sections from each level were stained with haematoxylin and eosin (H&E), DAPI and Masson's trichrome as previously described [26]. All sections were viewed using an upright Carl Zeiss Axio Imager. M2 incorporating an Axio Cam MRc5, which was controlled by Zen Pro software 2012 (Zeiss).

### 2.5 Determination of DNA content of native and decellularised pSFT

Samples of the ankle-, mid- and toe-region of each of 6 native pSFT and the 6 QA tendon halves were finely macerated and mixed together. Triplicate samples (approx. 100 mg native; 250 mg decellularised) of the mixed-region tissue were lyophilized to constant weight. DNA was extracted from the lyophilized tissue using the DNeasy Blood & Tissue Kit (Qiagen) and the concentration of DNA in the extracts was determined by Nanodrop spectrophotometry at 260 nm.

### 2.6 Determination of the biocompatibility of decellularised pSFT

Contact cytotoxicity assays were used to evaluate the biocompatibility of the 6 QA tendon halves as previously described [26]. Murine L929 fibroblasts and Baby Hamster kidney (BHK) cells were cultured in contact with samples (approximately 2 × 2 × 5 mm) of tendon from each tissue region, which were attached to the bottom of six well plates using Steri-Strip™ (3 M). A drop of cyanoacrylate contact adhesive served as the positive control for cytotoxicity and Steri-Strip (3 M; 10 × 3 mm) as the negative control. Two plates were created for each tendon, containing ankle, middle and toe regions of tissue, and cell only, positive and negative controls. The tissues and controls were incubated with L929 and BHK cells in an atmosphere of 5% (v/v) CO_2_ in air at 37 °C for 48 h. Samples were viewed using phase contrast microscopy on an inverted microscope (Olympus UK, model IX71) and images of each sample were captured digitally.

Cell monolayers were fixed in 10% (v/v) NBF for 10 min prior to staining with Giemsa stain. After rinsing off excess dye, samples were imaged again under bright field microscopy. The tissues were judged to be biocompatible if the cells grew up to and in contact with the edge of the samples.

### 2.7 Tensile testing of decellularised porcine superflexor tendons

**Fig. 1:**
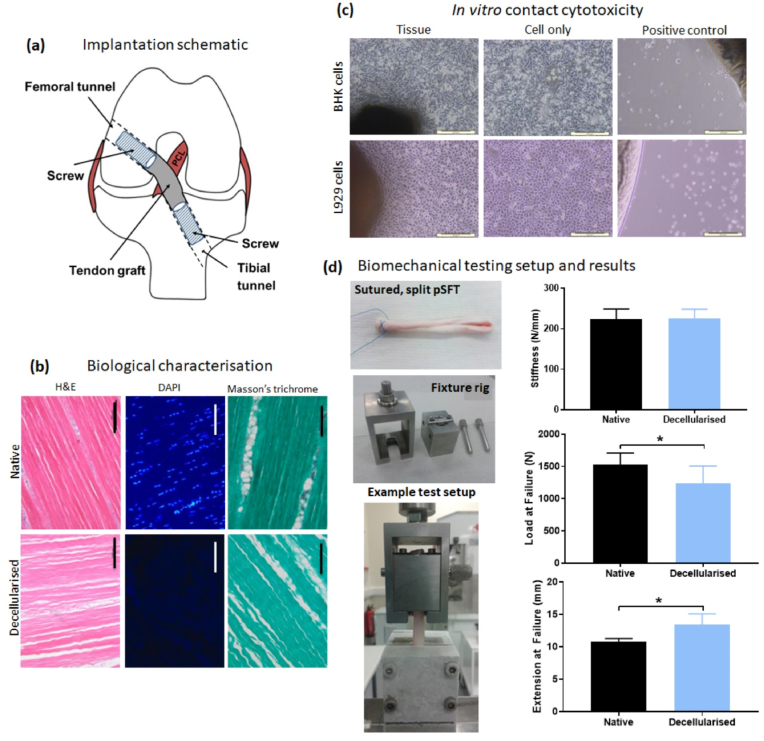
**Implantation schematic and quality assurance of decellularised pSFT for implantation.** A schematic of graft placement is provided for reference (a). Biological characterisation shows representative images (b) following staining of histological sections of native (top row) and decellularised (bottom row) tissues with H&E, DAPI and Masson's trichrome. The same exposure settings were used for DAPI images of fresh and decellularised pSFT. *In vitro* contact cytotoxicity testing (c) of decellularised pSFT using BHK (top row) and L929 (bottom row) cell lines. Representative images shown of pSFT samples, cell only wells and cyanoacrylate positive controls; scale bars show 200 μm. Biomechanical testing setup (d) demonstrates the sutured, split tendon, bespoke fixture rig and system clamped in place on the testing machine, as well as the stiffness, load at failure and extension at failure of native and decellularised tendons. Graphs show mean ± 95% confidence intervals. * denotes significant difference between groups (p < 0.05, upaired *t*-test).

Biomechanical properties of the QA tendon halves were obtained by simulating a *trans*-femoral fixation technique. The tendons were looped over a metal bar in a bespoke testing rig and sutured together at the opposite end (Fig. 1d). The lower end of the graft was fixed in place using a bespoke cryogrip filled with dry ice to prevent slippage during testing.

Testing was carried out using an Instron 3365 (Instron, Bucks, UK) materials testing machine equipped with a 1000 N load cell. Twelve preconditioning cycles (15 mm min^−1^, 0–50 N) were applied to the graft, followed by a ramp to failure at 30 mm min^−1^. Stiffness, failure load and failure extension were determined from the load curves. Native pSFT were split as for decellularised tissues and the mechanical properties determined using the same method.

### 2.8 *In vivo* performance of decellularised porcine superflexor tendons

The *in vivo* study was conducted by the NAMSA test facility (Chasse-sur-Rhone, France) in adaptation of the ISO 10993 Standard on “Biological evaluation of medical devices”, Part 6 (2007). The study was in accordance with European requirements (2010/63EU) under a protocol approved by the NAMSA ethical committee (2015). NAMSA is an accredited facility registered with the French Department of Agriculture for animal housing, care and investigations. Prior to commencement of this study, implantation parameters were established through several pilot studies by the surgical and research team. Cadaveric sheep and sheep under non-recovery anaesthesia underwent graft implantation to establish the most appropriate combination of graft, tunnel and interference screw dimensions for the ovine knee. The ACL of 38 sheep (female, Blanche du Massif Central; 46–86 kg; 1.9–3.8 years) was then removed and reconstructed with split, decellularised pSFT (test) or cellular oSFT (allograft control). After 4, 12 and 26 weeks of implantation, the behaviour of the implants and local tissue effects on the knee joint were macroscopically and histopathologically evaluated at NAMSA. After 26 weeks, the biomechanical performance of the pSFT and oSFT was evaluated at the University of Leeds. Details of the number of animals used for each group is presented in Table (2). The reserve sheep were operated upon using the same surgical procedure as the other sheep in case of adverse events and then followed in the same manner up to the end of the study. The number of animals in each group was the minimum required to evaluate the biological integration and biomechanical performance at 6 months based upon the literature [[33], [34], [35], [36], [37]].

**Table 2:** Details of the number of animals used during the study for clinical, histological and biomechanical analysis.

Time period	Number of animals	Macroscopic and histopathologic analysis		Macroscopic and biomechanical analysis			Clinical observations	
		pSFT	oSFT	pSFT	oSFT	Contralateral knees	pSFT	oSFT
4 weeks	8	4	4	0	0	0	18	18
12 weeks	8	4	4	0	0	0	14	14
26 weeks	20	4	4	6	6	6	10	10
Reserves	2	/	/	1	1	/	1	1

### 2.9 Surgical procedure and animal husbandry

Prior to surgery, pSFT and oSFT grafts were thawed in their packaging and transferred to sterile saline soaked gauze in the operating theatre. Grafts were trimmed to 11 cm length, 7 mm diameter and a passing suture (Ethibond® 1 or Ethibond® 2) placed at each end along for approximately 10–15 mm.

Sheep were weighed and premedicated (intravenous; IV) with diazepam (Valium®, Roche) and butorphanol (Torbugesic®, Pfizer or Torphasol®, Axience) and anaesthetised (IV) using propofol (Propovet®, Abbot). Sheep were intubated and mechanically ventilated. Anaesthesia was maintained by inhalation of oxygen-isoflurane (Iso-Flo®, Axience). Pre-operatively sheep received an intramuscular (IM) injection of a non-steroidal anti-inflammatory (flunixine, Meflosul®, Zoetis) and peri-operatively an antibiotic (IM; amoxicillin, Duphamox®, LA). Sheep were placed in dorsal recumbency and monitored throughout the procedure. Surgeries were performed by a veterinary surgeon. The joint was opened by a lateral arthrotomy and the ACL removed. The knee was then moved in the sagittal plane to verify the anterior laxity between the femur and tibia (drawer test). A tibial guide rail (Mitek) was hooked into the ACL tibial insertion and the angle was set at approximately 50–55°. A crossing tibial tunnel was drilled from the medial/proximal tibia to the tibial plateau, then over-drilled with an acorn reamer (Mitek) to a final diameter of 7 mm. The extra-articular and intra-articular edges of the tibial tunnel were softened with a 7 mm dilatator to prevent wear on the graft. The femoral tunnel was similarly prepared (angle 40–45°, tunnel diameter 8 mm). The passing suture placed on the test or control grafts was introduced through the tibial and femoral tunnels using a pin with an eyelet. The extra-articular edge of the femoral tunnel with the article was enlarged with a 7 mm dilator (approximately 10 mm deep) and a bioresorbable interference screw (Milagro BR, Mitek; 7 mm ø, 23 mm long) was manually inserted into the tunnel using a modular driver. The extra-articular edge of the tibial tunnel with the graft enlarged and the graft firmly held with a tie tensioner (5 lb) using the two passing sutures while the knee was maintained in a half-flexion position. An interference screw (Milagro BR, Mitek; 7 mm ø, 30 mm long) was then manually inserted in the tibial tunnel using a modular driver. Grafts were cut at the tunnel's exit and no passing suture remained in the bone tunnels. A schematic of the placement of the graft within tunnels is shown in Fig. 1a.

The tension of the articles was checked in hyperflexion and hyperextension before movement of the knee in the sagittal plane to verify the absence of laxity after ACL reconstruction. The joint capsule and the muscles were separately closed using discontinuous (simple interrupted) absorbable sutures (1 PDS™ II, Ethicon). The subcutaneous layer was closed using absorbable continuous suture (Vicryl® 2.0, Ethicon) and the skin using surgical staples (Covidien™). The surgical wound was disinfected with iodine solution (Vetedine® solution, Vetoquinol).

The sheep were kept in a sling for approximately 24 h after the surgery to minimize the risk of traumatic movement during the recovery from anaesthesia. While in the sling, the operated legs were moved at their full amplitude three to five times a day. After this period, the sheep were housed without restriction of movement (stifles loaded) individually or in a group in a limited space but always keeping social contact, under standardised laboratory conditions. Food and water were provided ad libitum. The operated legs were moved at their full amplitude once to twice a day during the first two weeks of the study. Buprenorphine was administered once to twice daily for 6 days post-surgery and flunixine (Meflosyl®, Pfizer) daily for 10 days. Every two days for 14 days, amoxicillin (Duphamox LA®, Pfizer) was administered and wounds disinfected with iodine solution (Vetedine® solution, Vetoquinol). Surgical staples were removed after complete healing (14 days after surgery).

### 2.10 Clinical follow-up

The sheep were observed daily. The gait (including assessment of flexion and extension through manual manipulation and observation of animals during walking on a flat surface), weight bearing, swelling of the site, stiffness of the leg and cracking at flexion was evaluated once a week during the first month and then every month until the end of the study. At 4, 12 or 26 weeks following surgery the designated sheep were weighed and euthanised by IV injection of pentobarbital. Qualitative macroscopic observations of the synovial fluid, graft and knee joint tissues were made at necropsy and images of the operated knee were captured. If an animal became injured, ill or moribund during the course of the study, care was conducted in accordance with current veterinary medical practices. When warranted for humane reasons, euthanasia was conducted following veterinary advice, a full necropsy was conducted to determine the cause of death and implanted sites were sampled and fixed in 10% (v/v) neutral buffered formalin for further histopathologic evaluation.

### 2.11 Cytological analysis of the synovial fluids

After smearing, the synovial fluids (n = 4 per group and time point) sampled at termination were stained with May-Grunwald Giemsa and a qualitative and semi-quantitative evaluation of the cellular components (monocytes, polymorphonuclear cells (PMNs), lymphocytes, synoviocytes) plus the presence of particulates was carried out. Cells were graded from 0 (absent); 1 (slight or 1–5 cells per field of view at 400× magnification), 2 moderate (or 6–10 cells per FoV), 3 (marked or heavy cell infiltrate) and 4 (severe or packed cells).

### 2.12 Histopathological evaluation of local tissue effects and integration

After 4, 12 or 26 weeks, sheep with ACL reconstructions comprising oSFT and pSFT (n = 4 per group) were sacrificed for macroscopic and histopathological evaluation of the grafts and surrounding tissues. Following euthanasia, mobility of the joint was assessed (drawer test, joint stiffness, swelling) prior to dissection of the joint. Extracapsular tissues were removed to allow visualisation of the graft and macroscopic evaluation was carried out to assess adverse effects on the joint. For histological analysis of the intra-articular graft, the central third of each oSFT and pSFT graft was extracted and fixed in 10% (v/v) NBF, prior to dehydration and embedding (longitudinal and transverse samples) in paraffin wax. Sections were cut and stained with safranin-haematoxylin-eosin (SHE). The two remaining pieces of graft within the joint space were snap frozen in liquid nitrogen and stored at −40 °C. Samples of the posterior cruciate ligament (PCL), meniscus, popliteal lymph node, synovial membrane and smears from synovial fluid of the operated knee were also collected and processed for histological evaluation. For histological evaluation of tissue integration in bone tunnels, femoral and tibial insertion sites were excised and fixed in 10% (v/v) NBF prior to dehydration and embedding in poly(methyl methacrylate) (PMMA). One longitudinal section of each was produced using an Exakt (Hamburg-Norderstedt, Germany) grinding system and stained with modified Paragon.

Histological sections were assessed for cellular infiltration, cell type, tissue remodelling and integration by a trained pathologist at NAMSA. A qualitative and semi-quantitative histopathologic evaluation of the local tissue effects was conducted based on an adaptation of ISO10993, “Biological evaluation of medical devices” Part 6 (2007). Sections of the graft tissue were graded from 0 (absent); 1 (slight or 1–5 cells per field of view at 400× magnification), 2 moderate (or 6–10 cells per FoV), 3 (marked or heavy cell infiltrate) and 4 (severe or packed cells) for PMNs, lymphocytes, plasma cells, macrophages and giant cells/osteoclastic cells and performance parameters. For sections of the bone tunnels, performance parameters were assessed using the same grading system with analysis of the presence of Sharpey's fibres, quality of the tendon-bone junction in the bone tunnels, osseointegration (between the interference screw and the native femoral/tibial bone), neovascularization and screw degradation.

### 2.13 Histological and immunohistochemical analysis of graft tissue

Remaining NBF fixed paraffin embedded tissue blocks and snap frozen tissue samples were returned to the University of Leeds for further assessment of the cellularity of the central regions of the graft tissue only. The tissue was thawed and fixed using zinc fixative (0.1 M tris; 3.2 mM calcium acetate [Thermo Fisher scientific], 27 mM zinc acetate [Sigma Aldrich], 37 mM zinc chloride [Fluka]; pH 7.2) for 48 h. The zinc fixed samples were processed automatically (Leica 11,020 tissue processor) and embedded in paraffin wax. For comparative purposes, native ovine ACL tissue sections were evaluated using four ovine ACLs from Blanche du Massif Central sheep aged 20 months (NAMSA) from animals which were euthanised as part of other studies not relating to musculoskeletal conditions.

Routine histological staining techniques (haematoxylin & eosin (H&E), DAPI, Masson's trichrome) were applied to NBF-fixed fixed tissue sections (12 μm). Sections (12 μm) of NBF and zinc fixed tissues were taken and labelled using a range of antibodies as detailed in Table 3. The Dako EnVision Detection System, Peroxidase/DAB (Agilent Technologies) was used for antibody detection. The primary antibodies, dilutions, method of antigen retrieval and positive control tissues were established in extensive preliminary studies. Isotype control antibodies (Dako, Agilent Technologies) and omission of the primary antibodies were used to verify antibody specificity and as negative controls. Images were captured using an upright Carl Zeiss Axio Imager. M2 incorporating an Axio Cam MRc5, which was controlled by Zen Pro software 2012 (Zeiss).

**Table 3:** **Details of antibodies used for immunohistochemical labelling of tissue sections.** Isotype and reagent controls were included for all test groups, as well as positive control ovine tissue sections. For tissues fixed with zinc fixative, no antigen retrieval methods were used. ‘Incubation’ column refers to the primary antibody incubation step. RT: room temperature. * Sections blocked for 30 min using 2% (v/v) milk in TBS immediately prior to application of primary antibody.

Antigen	Supplier	Product code	Working dilution	Isotype	Antigen retrieval	Incubation	Positive control	Antigen relevance
CD3	Leica	NCL-L-CD3-565	1:125	IgG1	N/A (zinc)	RT, 30 min	Sheep thymus	Pan T-cell marker
CD19	Leica	NCL-L-CD19-163	1:50	IgG2b	N/A (zinc)	RT, 60 min	Sheep lymph node	Pan B-cell marker
CD163	AbD Serotec	MCA1853	1:100	IgG1	Protease K (Dako Agilent), RT, 20 min	RT, 45 min	Sheep lymph node	Heamoglobin/haptoglobin scavenger receptor Macrophages/M2 macrophages [[40], [41], [42], [43], [44]]
CD80	AbD Serotec	MCA2436GA	1:25	IgG1	Protease K, RT, 20 min	RT, 45 min	Sheep lymph node	Co-stimulatory molecule B.71; M1 macrophages [43,[45], [46], [47]]
MAC387	AbD Serotec	MCA874GA	1:100	IgG1	Protease K, RT, 20 min	RT, 2 h	Sheep lymph node	Recently infiltrating macrophages [MRP 14] and PMNs [[48], [49], [50]]
α-SMA	Dako	M0851	1:100	IgG2a	Protease K, RT, 20 min	RT 60 min	Sheep pulmonary artery	Myofibroblast marker, observed during wound healing [51,52]
CD271	Biolegend	345,102	1:100	IgG1	Protease K, RT, 20 min	RT 60 min	Sheep aorta	LNGFR (Low affinity nerve growth factor) multipotent stromal cells [53]
CTGF	Abcam	Ab6992	1:100	IgG1	Protease K, RT, 20 min + Trypsin-EDTA (Sigma), 30 s	RT, 30 min	Sheep skin	Connective tissue growth factor; roles in cell adhesion, migration, proliferation, angiogenesis, tissue wound repair and fibrosis [54,55]
CD34	Abcam	Ab81289	1:500	Rabbit IgG	N/A (zinc)	37 °C, 1 h*	Sheep skin	Surface glycoprotein heamatopoietic stem cells; diverse progenitors [56,57]
Col III	Abcam	Ab7778	1:1000	Rabbit IgG	N/A (zinc)	37 °C, 1 h*	Sheep vessel	Collagen in tendon intrafasicular split matrix [58,59]
Tenascin-C	Abcam	Ab3970	1:50	IgG1	N/A (zinc)	4 °C, overnight*	Sheep tonsil	Expressed in healing tendons and bone [60,61]
Ki-67	Dako	M7240	1:150	IgG1	N/A (zinc)	37 °C, 30 min*	Sheep small intestine	Marker of cell proliferation

Sections were subject to semi-quantitative analysis. For cell markers the number of positive cells per high power field of view (FoV; 40× objective; 400× magnification) were counted and scored 0 for no cells; 1 (rare) for 1–5 cells; 2 (moderate) for 6–10 cells; 3 (marked) for a heavy infiltrate and 4 (packed) for packed cells. For matrix proteins (tenascin C and collagen III) sections were scored 0 (absent), 1 (slight), 2 (moderate), 3 (strong) and 4 (intense).

### 2.14 Biomechanical performance

Biomechanical testing of ovine knees was carried out 26 weeks following ACL reconstruction. Whole knees were excised and frozen following euthanasia and shipped to Leeds on dry ice. Prior to testing, knees were thawed and the ends of the femur and tibia potted in PMMA cement and stored at 4 °C overnight. All remaining excess tissue, including musculature, extracapsular ligaments and menisci was dissected from the joint and the lateral condyle removed. Knees were secured in an Instron 3365, the PCL cut before alignment of the graft and bone tunnels to the axis of loading, providing a worst-case loading scenario. Samples underwent ten preconditioning cycles (100 mm s^−1^, 0–20 N) prior to testing to failure (200 mm s^−1^). In addition to testing of pSFT and oSFT grafts following 26 weeks implantation, native ovine ACLs (contralateral knees from sheep used in the study; n = 6) were tested using the same loading protocol. To investigate whether the biomechanical performance improved by 26 weeks compared to immediately following implantation, split pSFT not used in the study were implanted into the cadaveric contralateral knees (pSFT(C); n = 6). These implantations were carried out using the same surgical tools and procedures and underwent biomechanical testing using the same loading protocol as all other groups.

### 2.15 Statistical analysis

Scores for semi-quantitative analysis of the cellularity of the grafts are expressed as mean (n = 4) ± SD. One-way ANOVA was used to compare biomechanical properties of native ACLs, decellularised pSFT in cadaveric knees (pSFT(C)) and oSFT and decellularised pSFT 26 weeks post implantation. Tukey's post-hoc analysis was used to assess the significance of differences between groups (p < 0.05).

## 3 Results

### 3.1 Quality assurance of decellularised tendons

None of the tendons tested showed any signs of microbial contamination. Observations of tissue sections stained with H&E demonstrated that the histoarchitecture of the pSFT had been maintained during decellularisation and there was no evidence of cells or cell nuclei remaining (Fig. 1b). DAPI stained tissue sections showed no evidence of whole nuclei or DNA, with the exception of some small fragments in endotendon regions (Fig. 1b). These results were consistent across the ankle-, middle- and toe regions of the grafts. Masson's trichrome stained tissue sections showed an absence of cytoplasmic material in the bulk of the tissues (Fig. 1b), with any fragments remaining contained in single patches of endotendon.

Biocompatibility testing showed no evidence of *in vitro* cytotoxicity from any region of any decellularised pSFT (Fig. 1c). Both BHK and L929 cells grew up to the edge of the tissue sections, with no observable differences in morphology compared to cells alone and no signs of the voids present around cyanoacrylate controls.

Fresh pSFT had a total DNA content of 785.5 ± 112.2 ng mg^−1^ (mean n = 6 ± 95% confidence limits) dry weight tissue. In decellularised tendons, this was reduced to 14.9 ± 7.1 ng mg^−1^ (mean n = 6) an average reduction of 98.1%.

There was no significant difference in the stiffness of split native pSFT compared with split decellularised pSFT (223.2 ± 24.3 N mm^−1^ vs 226.4 ± 21.0 N mm^−1^ respectively [mean n = 6 ± 95% confidence limits]; 6–7 mm diameter grafts; Fig. 1d; top graph). There was a significant (p < 0.05) reduction in the load at failure (1528 ± 173 N native vs 1238 ± 156 N decellularised; Fig. 1d; middle graph) and an increase in extension at failure (10.78 ± 0.48 mm native vs 13.42 ± 1.59 mm decellularised; Fig. 1d; bottom graph).

### 3.2 Post-operative clinical observations

On day 1, one sheep was found dead due to compression of the trachea by the whole body sling used to prevent weight bearing during the first 24 h. Following this incident, slings were modified to prevent any other animals becoming tangled in the supports. Two sheep of the 4 week time period (one oSFT and one pSFT) slightly lost weight (2 and 3% respectively), which was associated with absent to slight weight bearing for one sheep. All sheep gained weight between implantation and termination. One sheep was euthanised on day 6 due to continued lack of standing over two days. Traumatic injury; likely due to sheep in the same pen, was confirmed at necropsy.

Some minor abnormalities were observed during the follow-up at a distance from the implanted sites that were not considered to have impacted the study results. One sheep in the oSFT group showed delayed wound healing to day 72. The wound was treated but affected follow-up on this sheep. At termination (26 weeks), synovial fluid was slightly pink but the abnormality was not considered to have impacted the study results.

No abnormalities, other than those features frequently found following ligamentoplasty were observed during the clinical follow-up. No major differences were observed in weight bearing, gait, joint alignment, swelling or cracking at flexion between sheep implanted with oSFT and decellularised pSFT at any time point. The number of sheep displaying abnormalities in these at each time point is summarised in Table 4. The gait of the sheep was observed to be normal or almost normal from one week after the surgery.

**Table 4:** **Occurrence of macroscopic observations at termination**. Figures indicate number of sheep demonstrating parameter at observation with the exception of the Outerbridge classification which was scored as follows: 0 = normal cartilage; 1 = cartilage softening and swelling; 2 = Partial-thickness defect with fissures on the surface that do not reach subchondral bone or exceed 1.5 cm in diameter; 3 = Fissuring to the level of subchondral bone in an area with a diameter more than 1.5 cm; 4 = Exposed subchondral bone.

Parameters observed for sheep dedicated for histopathology and biomechanical analysis			4 weeks		12 weeks		26 weeks	
			Ovine SFT* **(n** = **4)**	Decell pSFT* **(n** = **4)**	Ovine SFT* **(n** = **4)**	Decell pSFT* **(n** = **4)**	Ovine SFT* **(n** = **11)**	Decell pSFT* **(n** = **11)**
Synovial fluid	Abnormal colour		1	4	1	1	3	3
	Abnormal clearness		1	3	3	3	3	3
	Abnormal viscosity		4	4	1	1	0	2
	Effusion (marked amount)		1	1	1	0	0	1
Graft	Taut in flexion and extension		0	0	3	1	3	2
	Taut in flexion and not visible in extension		0	0	0	0	2	2
	Slacked in extension (taut in flexion)		3	2	0	1	4	6
	Slacked in flexion and extension		1	2	1	2	2	1
	Broken		0	0	0	0	0	0
	Ravelling/signs of damage		3	2	2	1	1	0
Gait	Limping		1	2	1	1	1	3
Movement of the knee	Cracking		1	3	2	2	7	8
Knee	Swelling		3	3	3	2	0	0
	Antero-posterior laxity		1	0	1	0	1	0
Parameters observed for sheep dedicated for histopathology			/	/	/	/	Ovine SFT* **(n** = **4)**	Decell pSFT* **(n** = **4)**
Synovial membrane	Discoloration/red spots		2	4	3	0	0	0
Menisci	Tear		0	0	1	0	0	0
	Superficial tears		1	0	0	0	0	0
	Displacement		0	0	0	0	1	0
Patella	Erosion		0	0	0	0	1	0
	Outerbridge scores		0; 0; 0; 0	1; 0; 0; 0	0; 0; 0; 0	0; 0; 0; 0	0; 0; 2; 0	0; 0; 0; 0
Tibial plateau	Erosion		3	2	1	2	3	4
	Outerbridge scores	Lateral Medial	1; 1; 1; 0 1; 2; 2; 2	1; 1; 1; 0 2; 1; 2; 1	1; 0; 1; 1 1; 0; 1; 2	2; 1; 1; 1 0; 2; 1; 1	1; 1; 3; 0 2; 2; 3; 1	1; 1; 2; 0 2; 2; 1; 2
Femoral condyles	Erosion		1	0	1	1	2	1
	Outerbridge scores	Lateral Medial	0; 0; 0; 0 1; 2; 0; 0	0; 0; 0; 0 1; 0; 1; 0	0; 1; 0; 0 1; 1; 2; 1	0; 0; 1; 0 0; 2; 1; 0	0; 1; 2; 0 1; 1; 1; 1	0; 0; 2; 0 2; 1; 2; 0
Posterior cruciate ligament	Separated fibers		0	0	1	0	0	0

### 3.3 Macroscopic observations at termination

**Fig. 2:**
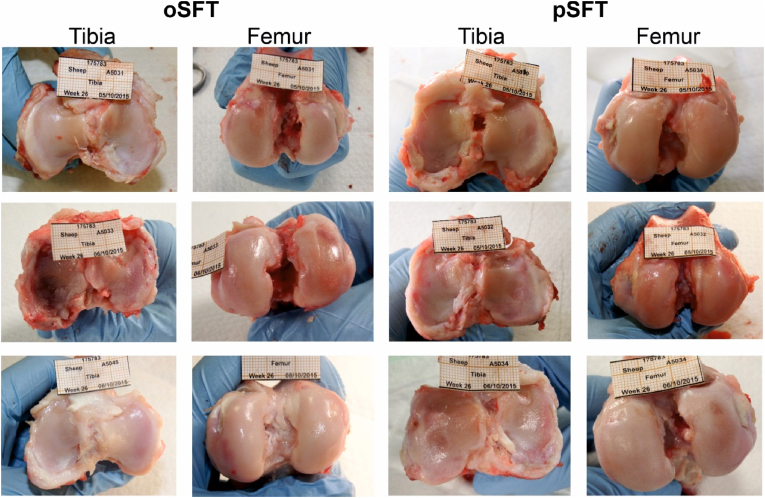
**Macroscopic images of tibial and femoral cartilage surfaces 26 weeks post implantation**. No difference in the prevalence of damage or lesions was observed between oSFT and pSFT groups. Images taken from ovine knee joints dissected for biological analysis.

At termination, post-operative complications such as knee swelling (only at 4 and 12 weeks), chondral erosion and graft slack occurred at similar incidences in both the decellularised pSFT and oSFT group (Table 4). Images of the macroscopic joint features at 26 weeks are provided in Fig. 2. Full details of the macroscopic observations at termination, together with images of each joint are openly available from the University of Leeds Data Repository [62].

### 3.4 Cytological evaluation of synovial fluids

The results of cytological evaluation of synovial smears are presented in Table (5). At 4 weeks, synovial smears from two sheep implanted with a pSFT and one from a sheep implanted with an oSFT suggested an intra-articular inflammatory process with moderate cellularity and presence of PMNs. The presence of infection could not be excluded. No significant cellular response was noted for any of the other synovial fluids. At 12 weeks, one synovial smear from a sheep implanted with an oSFT suggested an intra-articular inflammatory process with presence of a mixture of synoviocytes, PMNs, lymphocytes and monocytes. No significant cellular response was observed in the three other synovial fluids from sheep implanted with oSFTs. For the synovial fluids from sheep implanted with pSFT, all synovial smears were considered to be within normal limits. At 26 weeks, the level of inflammation observed in the synovial fluids was considered to be adverse only in one sheep implanted with a pSFT which showed an intra-articular inflammatory process with lymphocytes and PMNs and an infectious origin could not be excluded. There was no material suggestive of article residues or debris in any of the smears at any time point.

**Table 5:** **Summary of the semi-quantitative histopathologic evaluation of the synovial fluids**. After smearing, the synovial fluids (n = 4 per group and time period) sampled at termination were stained with May-Grünwald Giemsa (MGG). A qualitative and semi-quantitative evaluation of the cellular components (monocytes, PMNs, lymphocytes, synoviocytes) was conducted and graded from 0 (absent); 1 (slight or 1–5 cells per field of view at 400× magnification), 2 moderate (or 6–10 cells per FoV), 3 (marked or heavy cell infiltrate) and 4 (severe or packed cells). * The possibility of infection could not be ruled out for the explants from three sheep at 4 weeks (two decellularised pSFT and one ovine SFT) and from one sheep at 26 weeks (decellularised pSFT).

	4 Weeks		12 Weeks		26 Weeks	
	Decell pSFT*	Ovine SFT*	Decell pSFT	Ovine SFT	Decell pSFT*	Ovine SFT
Monocytes	0.5 ± 0.5	0.5 ± 0.5	0.3 ± 0.4	1.0 ± 0.0	0.3 ± 0.4	0.0 ± 0.0
Polymorphonuclear cells	1.3 ± 1.3	0.8 ± 0.4	0.3 ± 0.4	1.0 ± 0.0	0.3 ± 0.4	0.0 ± 0.0
Lymphocytes	0.8 ± 0.4	0.8 ± 0.4	0.5 ± 0.5	1.0 ± 0.0	1.0 ± 1.0	0.3 ± 0.0
Synoviocytes	1.0 ± 0.0	1.3 ± 0.4	1.0 ± 0.0	1.5 ± 0.9	1.8 ± 0.8	1.3 ± 0.0
Cellularity	2 slight 2 mod*	2 rare 1 slight 1 marked*	3 rare 1 slight	2 rare 1 slight 1 mod	2 rare 1 mod 1 marked*	3 rare 1 slight

### 3.5 Histolopathological evaluation of local tissue effects and integration

The complete pathology report including the semi-quantitative analysis of each graft tissue, synovial membrane, menisci, PCL, popliteal lymph nodes and synovial is openly available in the University of Leeds Data Repository [62]. A summary of the semi-quantitative histopathologic analysis of the femoral bone tunnels and intra-articular grafts is presented in Tables (6) and (7) respectively. The analysis of the tibial bone tunnels was similar to the femoral bone tunnels (data not shown).

**Fig. 3:**
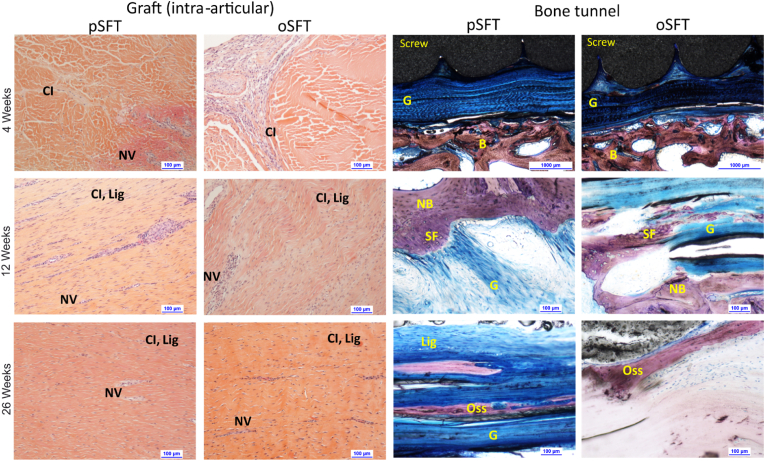
**Images of sections of the intra-articular graft portion (left) stained with SHE showing cellular infiltration and of the graft within bone tunnels (right) stained with modified Paragon showing graft integration.** Grafts showed evidence of cellular infiltration (**CI**), neovascularization (**NV**) at 4 weeks and ligamentisation (**Lig**) after 12 and 26 weeks. Within bone tunnels, evidence of cellular infiltration at the graft (**G**)- bone (**B**) junction could be seen at 4 weeks. By 12 weeks, there was evidence of new bone formation (**NB**) and Sharpey's fibres bridging the bone-tendon interface (**SF**). Evidence of ligament ossification (**Oss**) was observed at 26 weeks. Representative images shown, scale bars show 100 μm (except 4 weeks, bone tunnels, 1000 μm).

Representative images of cellular infiltration in the intra-articular grafts and integration of the grafts within the bone tunnels are presented in Figure (3). After 4 weeks, the two decellularised pSFT and one oSFT showing moderate to marked cellularity in synovial smears also had moderate to marked signs of inflammation in the intra-osseus and intra-articular parts of the graft (Table 6; Table 7). Overall, at 4 weeks, histopathological analysis indicated an ongoing inflammatory response in the bone tunnels (Table 6) and grafts (Table 7) comprising PMNs, lymphocytes, plasma cells and macrophages with some evidence of slight necrosis. Nevertheless, both the decellularised pSFT and oSFT showed evidence of peripheral cellular ingrowth in the intra-articular part of the graft and intra-osseous sites (Fig. 3; Table 6). The interface between the graft and the bone showed very slight signs of calcification and ossification, with moderate signs of osseointegration of the interference screw (Table 6). Overall, there were no relevant differences between the two groups.

**Table 6:** Summary of the semi-quantitative histopathologic evaluation of the femoral bone tunnels. Qualitative and semi-quantitative evaluation of the different components was conducted as follows: Fibrin, necrosis, osteolysis, ligament calcification, ligament ossification, fibrous encapsulation and necrotic bone debris were graded from 0 (absent); 1 (slight); 2 (moderate); 3 (marked) and 4 (severe). Cellular components (PMNs, lymphocytes, plasma cells, macrophages and giant/osteoclastic cells) were graded from 0 (absent); 1 (slight or 1–5 cells per field of view at 400× magnification), 2 moderate (or 6–10 cells per FoV), 3 (marked or heavy cell infiltrate) and 4 (severe or packed cells). Fibrosis was graded 0 (absent); 1 (narrow band); 2 (moderately thick band); 3 (thick band) and 4 (extensive band with signs of encapsulation). Fatty infiltrate and bone marrow was graded 0 (absent); 1 (minimal amount associated with fibrosis); 2 (several layers of fat and fibrosis); 3 (elongated and broad accumulation of fat cells around implant site) and 4 (extensive fat completely surrounding the implant). Finally, neovascularization was graded 0 (absent); 1 (minimal capillary proliferation foci, 1–3 buds); 2 (groups of 4–7 capillaries with supporting fibroblastic structures); 3 (broad band of capillaries with supporting fibroblastic structures) and 4 (extensive band of capillaries with supporting fibroblastic structures).

Time period	Group/Article	Animal number	Fibrin	Polymorphonuclear cells	Lymphocytes	Plasma cells	Macrophages	Giant cells/osteoclastic cells	Necrosis	Osteolysis	Fibrosis	Fatty infiltrate/bone marrow	Neovascularization	Ligament calcification	Ligament ossification	Fibrous encapsulation	Necrotic bone debris	Ligament Sharpeys fibers	Quality of the ACL bone junction	Screw osseointegration	Cellular ingrowth	Ligamentisation	Ligament degradation	Screw degradation
4 weeks	Test (n = 4)	**Mean**	**0.0**	**1.3**	**2.3**	**1.8**	**2.0**	**1.0**	**0.5**	**1.0**	**1.0**	**0.0**	**1.0**	**0.5**	**0.5**	**0.0**	**0.8**	**0.0**	**1.0**	**2.0**	**1.0**	**1.0**	**0.0**	**1.0**
		*SD*	*0.0*	*0.4*	*0.4*	*0.4*	*0.0*	*0.0*	*0.5*	*0.0*	*0.0*	*0.0*	*0.0*	*0.5*	*0.5*	*0.0*	*0.4*	*0.0*	*0.0*	*0.7*	*0.0*	*0.0*	*0.0*	*0.0*
	Control (n = 4)	**Mean**	**0.5**	**1.5**	**2.3**	**1.3**	**1.5**	**1.0**	**0.3**	**1.0**	**1.0**	**0.3**	**1.0**	**0.5**	**0.5**	**0.0**	**0.3**	**0.0**	**1.3**	**1.8**	**1.0**	**1.0**	**0.0**	**1.0**
		*SD*	*0.5*	*0.9*	*0.4*	*0.4*	*0.5*	*0.0*	*0.4*	*0.0*	*0.0*	*0.4*	*0.0*	*0.5*	*0.5*	*0.0*	*0.4*	*0.0*	*0.4*	*0.8*	*0.0*	*0.0*	*0.0*	*0.0*
12 weeks	Test (n = 4)	**Mean**	**0.0**	**0.8**	**1.3**	**1.0**	**1.0**	**1.0**	**0.0**	**0.3**	**1.3**	**0.0**	**2.0**	**1.8**	**1.8**	**0.0**	**0.0**	**1.8**	**2.3**	**2.8**	**2.3**	**2.5**	**0.0**	**1.0**
		*SD*	*0.0*	*0.8*	*0.4*	*0.0*	*0.0*	*0.0*	*0.0*	*0.4*	*0.4*	*0.0*	*0.0*	*0.4*	*0.4*	*0.0*	*0.0*	*0.8*	*0.4*	*0.4*	*0.8*	*0.5*	*0.0*	*0.0*
	Control (n = 4)	**Mean**	**0.3**	**0.5**	**1.0**	**0.3**	**1.0**	**1.0**	**0.0**	**0.0**	**1.0**	**0.0**	**2.0**	**1.8**	**1.8**	**0.0**	**0.0**	**1.3**	**2.5**	**2.5**	**1.8**	**1.8**	**0.0**	**1.0**
		*SD*	*0.4*	*0.5*	*0.0*	*0.4*	*0.0*	*0.0*	*0.0*	*0.0*	*0.0*	*0.0*	*0.0*	*0.4*	*0.4*	*0.0*	*0.0*	*0.4*	*0.5*	*0.5*	*0.4*	*0.4*	*0.0*	*0.0*
26 weeks	Test (n = 4)	**Mean**	**0.3**	**1.3**	**1.8**	**1.0**	**1.3**	**1.0**	**0.0**	**1.0**	**1.3**	**0.5**	**1.3**	**1.8**	**1.8**	**0.0**	**0.0**	**2.0**	**2.3**	**3.0**	**2.3**	**2.0**	**0.5**	**1.0**
		*SD*	*0.4*	*0.4*	*0.4*	*0.0*	*0.4*	*0.0*	*0.0*	*0.7*	*0.4*	*0.9*	*0.4*	*0.4*	*0.4*	*0.0*	*0.0*	*1.2*	*0.8*	*0.7*	*0.8*	*0.7*	*0.9*	*0.0*
	Control (n = 4)	**Mean**	**0.0**	**0.3**	**1.3**	**1.0**	**1.0**	**0.8**	**0.0**	**0.8**	**1.0**	**0.3**	**1.8**	**1.5**	**1.5**	**0.0**	**0.0**	**1.0**	**2.3**	**2.3**	**2.3**	**2.3**	**0.0**	**1.3**
		*SD*	*0.0*	*0.4*	*0.4*	*0.0*	*0.0*	*0.4*	*0.0*	*0.4*	*0.0*	*0.4*	*0.4*	*0.9*	*0.9*	*0.0*	*0.0*	*0.0*	*0.8*	*1.3*	*1.1*	*0.4*	*0.0*	*0.4*

**Table 7:** Summary of the semi-quantitative histopathologic evaluation of the intra-articular grafts. Qualitative and semi-quantitative evaluation of the different components was conducted as follows: Fibrin, necrosis, calcification, ossification, fibrous encapsulation, fibroplasia, haemorrhage, cellular ingrowth, ligamentisation, synovial sheath formation and ligament degradation were graded from 0 (absent); 1 (slight); 2 (moderate); 3 (marked) and 4 (severe). Cellular components (PMNs, lymphocytes, plasma cells, macrophages and giant cells) were graded from 0 (absent); 1 (slight or 1–5 cells per field of view at 400× magnification), 2 moderate (or 6–10 cells per FoV), 3 (marked or heavy cell infiltrate) and 4 (severe or packed cells). Fibrosis was graded 0 (absent); 1 (narrow band); 2 (moderately thick band); 3 (thick band) and 4 (extensive band with signs of encapsulation). Fatty infiltrate was graded 0 (absent); 1 (minimal amount associated with fibrosis); 2 (several layers of fat and fibrosis); 3 (elongated and broad accumulation of fat cells around implant site) and 4 (extensive fat completely surrounding the implant). Finally, neovascularization was graded 0 (absent); 1 (minimal capillary proliferation foci, 1–3 buds); 2 (groups of 4–7 capillaries with supporting fibroblastic structures); 3 (broad band of capillaries with supporting fibroblastic structures) and 4 (extensive band of capillaries with supporting fibroblastic structures).

Time period	Group/Article	Animal number	Fibrin	Polymorphonuclear cells	Lymphocytes	Plasma cells	Macrophages	Giant cells	Necrosis	Fibrosis	Fatty infiltrate	Calcification	Ossification	Fibrous encapsulation	Fibroplasia	Neovascularization	Haemorrhage	Cellular ingrowth	Ligamentisation	Synovial sheath formation	Ligament degradation
4 weeks	Test (n = 4)	**Mean**	**0.8**	**1.5**	**1.3**	**0.8**	**1.5**	**0.5**	**0.8**	**0.0**	**0.0**	**0.3**	**0.3**	**0.0**	**1.3**	**1.0**	**0.8**	**1.5**	**1.0**	**0.5**	**0.8**
		*SD*	*0.8*	*1.1*	*0.4*	*0.4*	*0.5*	*0.5*	*0.8*	*0.0*	*0.0*	*0.4*	*0.4*	*0.0*	*0.4*	*0.0*	*0.4*	*0.5*	*0.0*	*0.5*	*0.8*
	Control (n = 4)	**Mean**	**0.3**	**1.0**	**1.5**	**1.0**	**1.5**	**0.3**	**0.0**	**0.0**	**0.3**	**0.0**	**0.0**	**0.0**	**1.0**	**1.0**	**0.3**	**1.5**	**0.5**	**1.0**	**0.0**
		*SD*	*0.4*	*0.7*	*0.5*	*0.7*	*0.5*	*0.4*	*0.0*	*0.0*	*0.4*	*0.0*	*0.0*	*0.0*	*0.0*	*1.0*	*0.4*	*0.5*	*0.5*	*0.7*	*0.0*
12 weeks	Test (n = 4)	**Mean**	**0.3**	**0.8**	**1.8**	**0.8**	**1.8**	**0.3**	**0.0**	**0.0**	**0.0**	**0.0**	**0.0**	**0.0**	**0.3**	**1.8**	**0.5**	**3.3**	**3.0**	**2.0**	**0.0**
		*SD*	*0.4*	*0.4*	*0.4*	*0.4*	*0.4*	*0.4*	*0.0*	*0.0*	*0.0*	*0.0*	*0.0*	*0.0*	*0.4*	*0.4*	*0.5*	*0.8*	*0.7*	*0.7*	*0.0*
	Control (n = 4)	**Mean**	**0.3**	**0.3**	**1.0**	**0.3**	**1.0**	**0.0**	**0.0**	**0.0**	**0.0**	**0.0**	**0.0**	**0.0**	**1.0**	**1.5**	**0.5**	**3.8**	**1.8**	**2.5**	**0.5**
		*SD*	*0.4*	*0.4*	*0.0*	*0.4*	*0.0*	*0.0*	*0.0*	*0.0*	*0.0*	*0.0*	*0.0*	*0.0*	*0.7*	*0.5*	*0.5*	*0.4*	*0.4*	*0.9*	*0.9*
26 weeks	Test (n = 4)	**Mean**	**0.3**	**0.5**	**1.3**	**0.5**	**1.3**	**0.0**	**0.3**	**0.0**	**0.0**	**0.0**	**0.0**	**0.0**	**0.3**	**1.5**	**0.0**	**3.3**	**3.3**	**2.3**	**0.5**
		*SD*	*0.4*	*0.5*	*0.8*	*0.5*	*0.8*	*0.0*	*0.4*	*0.0*	*0.0*	*0.0*	*0.0*	*0.0*	*0.4*	*0.9*	*0.0*	*0.8*	*0.8*	*0.4*	*0.9*
	Control (n = 4)	**Mean**	**0.0**	**0.0**	**0.5**	**0.0**	**0.5**	**0.0**	**0.0**	**0.0**	**0.0**	**0.3**	**0.0**	**0.0**	**0.0**	**0.8**	**0.0**	**4.0**	**3.5**	**2.0**	**0.0**
		*SD*	*0.0*	*0.0*	*0.5*	*0.0*	*0.5*	*0.0*	*0.0*	*0.0*	*0.0*	*0.4*	*0.0*	*0.0*	*0.0*	*0.4*	*0.0*	*0.0*	*0.9*	*0.7*	*0.0*

After 12 weeks, the overall severity of the local tissue effects was decreased and performance parameters increased for both groups when compared with the 4 week results. Both the decellularised pSFT and oSFT showed evidence of marked cellular ingrowth in the intra-articular graft and within the bone tunnels (Fig. 3; Table 6, Table 7). Moderate to marked ligamentisation was evident in the intra-articular graft sections (Fig. 3; Table 7). The interface between the graft and the bone showed slight to moderate signs of calcification and ossification, formation of Sharpey's fibers, and ACL/bone junctions of moderate quality (Table 6). When compared with the oSFT group, decellularised pSFTs had slightly higher mean scores of inflammatory cells but slightly higher mean scores of ligamentisation in both the intra-articular (Table 7) and intra-osseous parts of the grafts (Table 6). In view of the small size of the groups, these slight differences were not considered to be quantitatively relevant.

After 26 weeks, there were no major changes in local tissue effects and performance parameters when compared with the 12-week period. The decellularised pSFT had slightly higher mean scores of inflammatory cells compared with oSFT, however, infection could not ruled out for one of these grafts. This did not appear to impact on the graft performance, which was similar for both groups.

### 3.6 Histological and immunohistochemical analysis of intra-articular graft and native ovine ACL tissues

**Fig. 4:**
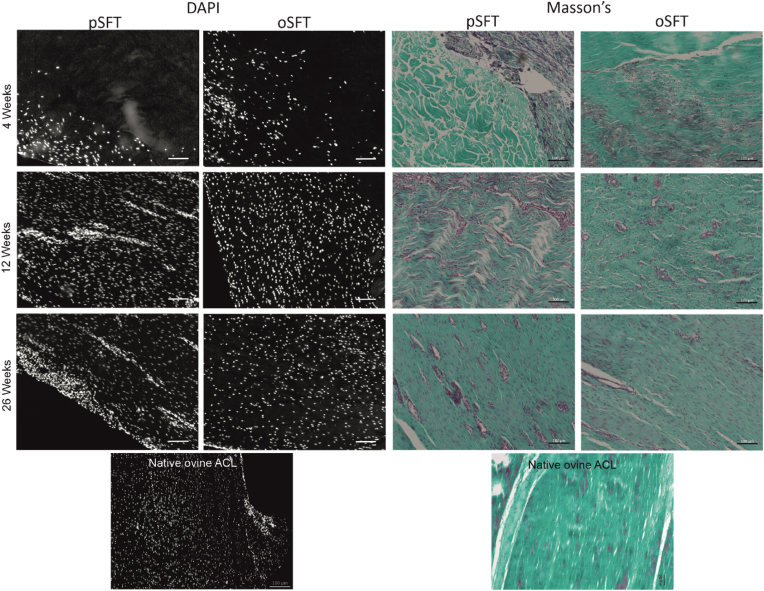
**DAPI and Masson's trichrome stained sections of implanted grafts at each time point**. Cellular infiltration was observed from 4 weeks, with evidence of vascularisation and further colonisation at 12 and 26 weeks for both pSFT and oSFT. Images are also provided for tissue from the native ovine ACL. Representative images shown, scale bars show 100 μm.

**Fig. 5:**
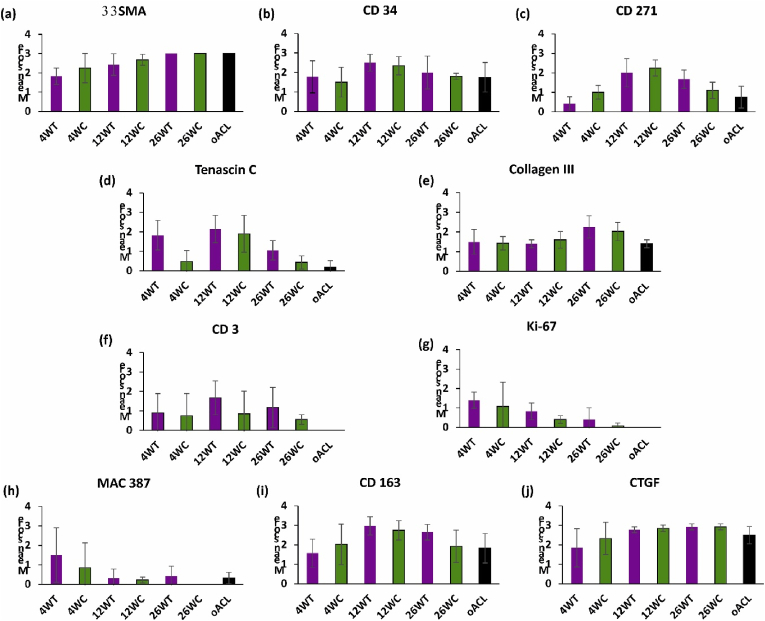
**Semi-quantitative analysis of the immunohistologically stained sections of implanted grafts and native ovine ACL at each time point.** For cell markers the number of positive cells per high power field of view (40× objective; 400× magnification) were counted and scored: 0 for no cells; 1 (rare) for 1–5 cells; 2 (moderate) for 6–10 cells; 3 (marked) for a heavy infiltrate and 4 (packed) for packed cells. For matrix proteins (tenascin C and collagen III) sections were scored 0 (absent), 1 (slight) 2 (moderate), 3 (strong) and 4 (intense). Data are presented as mean (n = 4) ± SD.

**Fig. 6:**
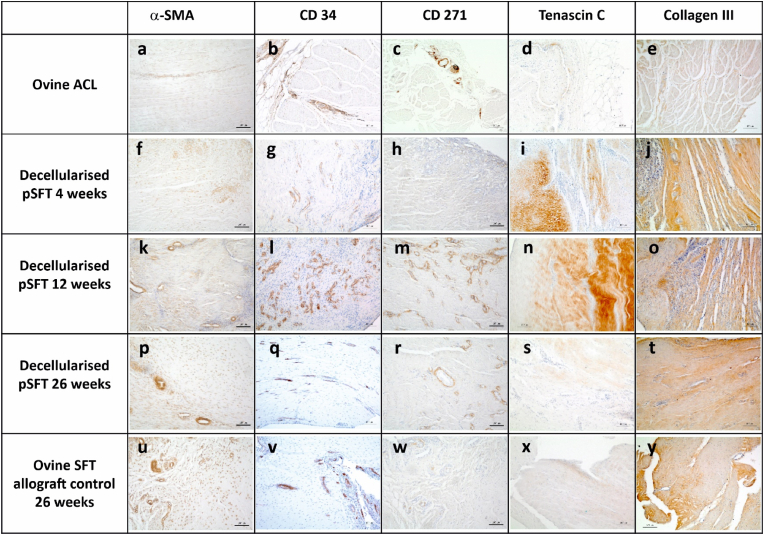
**Representative images of sections of the native ovine ACL and intra-articular graft portion stained with antibodies to α-SMA, CD 34, CD271, Tenascin C and collagen III.** A-E Native ovine ACL; F-J decellularised pSFT explanted at 4 weeks; K–O; decellularised pSFT explanted at 12 weeks; P-T decellularised pSFT explanted at 26 weeks; U–Y ovine SFT (allograft control) explanted at 26 weeks. Images captured at 10× magnification unless otherwise stated. Scale bars show 100 μm.

**Fig. 7:**
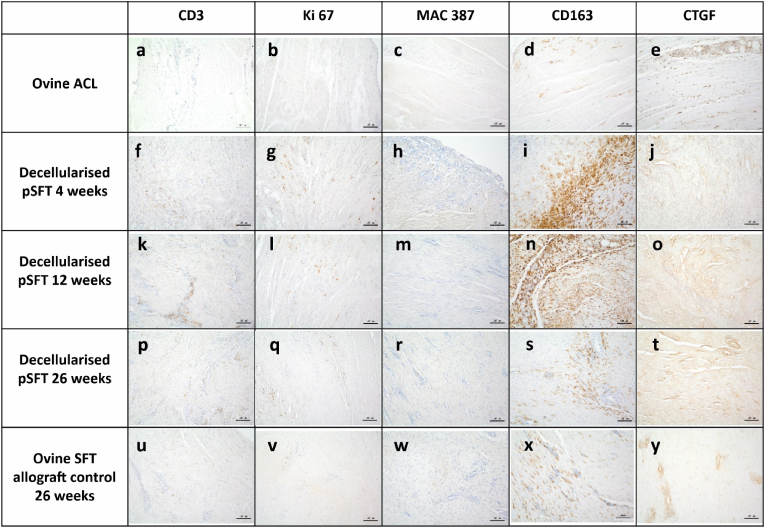
***Representative images of sections of the native ovine ACL and intra-articular graft portion stained with antibodies to CD3, Ki-67, MAC 387; CD163 and* CTGF.** A-E Native ovine ACL; F-J decellularised pSFT explanted at 4 weeks; K–O; decellularised pSFT explanted at 12 weeks; P-T decellularised pSFT explanted at 26 *weeks; U–Y ovine SFT (allograft control) explanted at 26 weeks. Images captured at 10× magnification unless otherwise stated. Scale bars show* 100 μm.

Sections of native ovine ACLs and grafts explanted at each time-point were stained with H&E, DAPI and Masson's trichrome to evaluate the cellularity and overall tissue structure. Representative images of sections stained with DAPI and Masson's (H&E not shown) are presented in Figure (4). Analysis of these tissue sections supported the histopathological observations of extent of inflammation, cellular ingrowth, vascularisation and ligamentisation of both the decellularised pSFT and oSFT tissues over time, which was similar for both groups. Data on the semi-quantitative evaluation of the immunohistochemical analysis of the matrix and cells populating the native ovine ACL and explanted tissues are presented in Figure (5) and representative images presented in Fig. 6, Fig. 7.

The majority of the stromal cells in sections of native ovine ACL stained weakly positive for α-SMA (Figs. 5a and 6a). Strongly stained α-SMA + cells were present in the walls of the vascular structures and the vascular endothelium stained positive with antibodies to CD34 (Fig. 6b). A variable proportion of the elongated stromal cells along the collagen fibres stained positive for CD34 (Fig. 6b). CD271+ cells were specifically located surrounding vascular structures, presumably representing pericytes (Fig. 6c). The extracellular matrix of the ovine ACLs was negative for tenascin-C (Figs. 5d and 6d). There was mild, but clear staining of the ligament collagen fibrils with antibodies to collagen III (Figs. 5e and 6e). There were no CD3^+^ (Figs. 5f and 7a), Ki-67+ (Figs. 5g and 7b), CD19^+^ or CD80^+^ (data not shown) cells in the native ACL tissues. Rare MAC 387+ cells were found in the endotenon or synovial sheath (Figs. 5h and 7c). A significant proportion of the cells in the ovine ACL tissue sections were CD163+, located in the synovial sheath and endotenon regions with an irregular or rounded morphology with occasional CD163+ cells elongated along the collagen fibrils (Figs. 5i and 7d). A considerable proportion (circa 50%) of the cells present throughout the ovine ACL tissues expressed CTGF (Figs. 5j and 7e).

Immunohistochemical analysis of sections of the intra-articular portion of explanted decellularised pSFT and oSFT revealed a similar pattern (Fig. 5). At 4 weeks large numbers of α-SMA + cells were present at the margins of the tissues between the collagen fibre bundles and along collagen fibres (Figs. 5a and 6f). The outer margins of the tissues contained numerous vascular structures, demarcated by α-SMA + cells (Fig. 6f). CD34^+^ cells were localised to the vascular structures (Figs. 5b and 6g). There were sparse CD271+ cells (Figs. 5c and 6h). There was mild to strong staining for tenascin-C (Fig 5d; Fig 6i) and collagen III (Fig 5e; Fig 6j) along the collagen fibres in the areas of cellular population. Variable numbers of CD3+cells (Figs. 5f and 7f) and Ki-67+ cells (Figs. 5g and 7g) were present, largely in inflammatory foci in the margins all of the explanted tissues indicating an ongoing inflammatory response. There were high numbers of MAC 387+ cells (grade 3–4) in tissue sections from the three sheep (2 decellularised pSFT, 1 oSFT) which showed moderate/marked cellularity in synovial smears. These were most likely PMNs as judged by histology. Rare MAC 387+ cells were present in the remaining tissues (Figs. 5h and 7h). CD163+ cells were abundant in the cellular infiltrate at the margins of the tissues (Fig. 5i). Indeed, CD163+ cells appeared to be the “pioneer” cells at the interface between the pSFT tissue that was decellularised and the tissue that was populated by cells (Fig. 7i). A high proportion of the cells populating the tissues were positive for CTGF (Figs. 5j and 7j).

After 12-weeks implantation, moderate to marked numbers of α-SMA + cells (Figs. 5a and 6k) were present in the central areas of the tissues. Abundant vascular structures were marked by CD34^+^ cells (Figs. 5b and 6l). Numbers of CD271+ cells increased, in a perivascular location (Figs. 5c and 6m). The mild to strong staining for tenascin-C (Fig 5d; Fig 6n) and collagen III (Fig 5e; Fig 6o) along the collagen fibres was still present. CD3^+^ cells were present in a perivascular location throughout the tissues (Figs. 5f and 7k). Ki-67+ cells (Figs. 5g and 7l) and MAC 387+ cells (Figs. 5h and 7m) were rare. Moderate to marked numbers of CD163+ cells had penetrated the central areas of the tissues (Figs. 5i and 7n). A high proportion of the cells were positive for CTGF (Figs. 5j and 7o).

At 26 weeks, marked numbers of the stromal cells were α-SMA+ (Figs. 5a, 6p and 5u). Overall there appeared to be a reduction in vascularisation, indicated by a slight reduction in CD34^+^ cells (Figs. 5b, 6q and 6v) and CD271+ (Figs. 5c, 6r and 6w) cells compared to 12-weeks. At 26 weeks, the numbers α-SMA, CD34 and CD271 positive cells were similar to those in the native oACL tissue sections (Fig. 5a–c). Tenascin C staining had reduced to mild compared to 4 and 12 weeks (Fig 5d; Fig 6s; Fig 6x) whilst collagen III staining increased to moderate/strong (Fig 5e; Fig 6t; Fig 6y). The tissue sections all showed the presence of some CD3^+^ cells, however the numbers were reduced compared with the 4 and 12-week explants (Figs. 5f, 7p and 7u). Ki-67+ (Figs. 5g, 7q and 7v) and MAC 387+ cells (Figs. 5h, 7r and 7w) were absent/rare. Although still present in moderate numbers, numbers of CD163+ cells were reduced (Fig. 5i). The CD163+ cells were present in vascularised areas, in the outer sheaf and also as elongated cells along the collagen fibrils (Fig. 7s and x) in numbers similar to the native ovine ACL (Fig. 5i). A high proportion of the cells were positive for CTGF (Figs. 5j and 7t; Fig 7y), similar to native ovine ACL tissues.

All tissue sections at 4, 12 and 26 weeks from the decellularised pSFT and oSFT were devoid of CD80^+^ and CD 19+ cells, only the occasional positive cell was identified in the inflammatory infiltrate of tissue from the one 26-week decellularised pSFT which was suspected of having an infection.

### 3.7 Biomechanical function of oSFT and pSFT ACL replacement

**Fig. 8:**
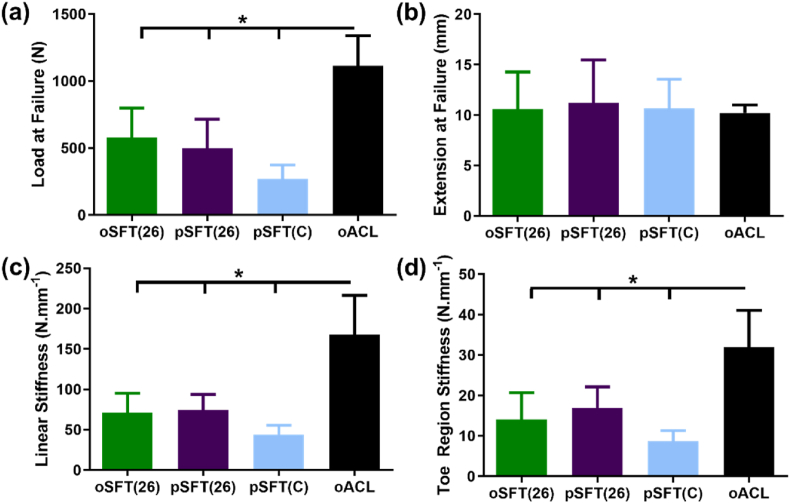
**Biomechanical properties of native and reconstructed ovine ACLs.** Graphs show mean ± 95% confidence intervals. * denotes significant difference between groups (1-way ANOVA; p < 0.05, Tukey's post hoc analysis).

Biomechanical properties of reconstructed ovine ACLs (oSFT and pSFT at 26 weeks, pSFT(C)) are presented in Fig. 8, along with the properties of native ovine ACLs from contralateral knees. There were no significant differences between the properties of the pSFT and oSFT at 26 weeks. There were no significant differences between the extension at failure for any group. All reconstructed ACLs showed a significantly lower (p < 0.05) linear stiffness compared to native ACL, with no further differences between groups. The load at failure for all reconstructed ACLs was significantly lower (p < 0.05) compared to the native ACL. In all native ACL, oSFT and pSFT at 26 weeks, failure mode was intraligamentous, with the majority of failures occurring mid-ligament and the rest towards the femoral or tibial insertion but within the graft material (native ACL 4/6 mid-ligament, 2/6 femoral side; oSFT 5/6 mid-ligament, 1/6 tibial side; pSFT 4/6 mid-ligament, 2/6 femoral side). No failures were observed at the fixation points for the pSFT or oSFT grafts at 26 weeks. By comparison, for the pSFT implanted into cadaveric contralateral knees from the study animals (pSFT(C), all failures occurred due to failure at the interface and pull out of the graft from the tunnel (4/6 tibial insertion, 2/6 insertion).

## 4 Discussion

The primary purpose of this study was to evaluate the biological integration and mechanical performance of the decellularised pSFT in the sheep knee over a 6 month period. It was clearly demonstrated that the decellularised pSFT showed equivalent *in vivo*, biological and biomechanical performance compared to a cellular ovine SFT allograft control over a six month period.

Decellularised pSFT for implantation were rigorously produced using a batch quality assurance approach. This demonstrated removal of cellular material and the majority of the DNA with mean total DNA content 14.9 ± 7.1 ng mg^−1^, well below the suggested acceptable levels for biological scaffolds of 50 ng double stranded DNA per mg dry weight [14]. As reported previously [26] the use of low concentration SDS plus proteinase inhibitors enabled retention of the decellularised pSFT extracellular matrix structure. The failure load of the split decellularised pSFT extracellular matrix scaffolds (1238 ± 256 N) was comparable to the native ovine ACL from the literature (1200 ± 190 N [63]) and deemed suitable for reconstruction in this model. Although this is lower than the human ACL (2160 ± 157 N [64]), it is important to note that the cross sectional area of both the ovine ACL (20 ± 4 mm^2^ [63]) and split pSFT (32.3 ± 5.9 mm^2^) are lower than the human ACL (47 ± 13 mm^2^ [65]). As the strength of a structure is dependent on cross-sectional area as well as the material properties, it is not possible to directly compare the results obtained in this study to the human ACL.

Skeletally mature sheep were selected for the study because of the similarity of the knee to humans, allowing anatomic fixation. The main limitation of this model is that the ovine knee is smaller than the human and therefore requires smaller graft materials. Prior to commencing surgeries on living animals, several pilot studies were conducted in cadaveric sheep and sheep under non-recovery anaesthesia. The surgical technique, in terms of dimensions of the bone tunnels and the screws was investigated with input from the surgeon and research team. Due to the small size of the ovine stifle and high density of the cancellous bone, fixation procedures were modified slightly compared with human surgical fixation. Graft diameter was reduced to a range of 6–7 mm as larger grafts (and therefore tunnels) would have resulted in damage to the menisci. The relative size of the interference screw compared with the bone tunnel was also decreased, enabling stable graft fixation despite the density of the graft material. A one-month pilot study in living animals demonstrated that the final method of fixation was suitable.

Sheep implanted with ovine SFT allografts were used as controls, since tendon allografts are used clinically and the performance of tendon allografts as ACL replacements in sheep has been widely reported [34,36,66,67]. Overall, both types of graft showed good *in vivo* performance with no major differences observed during the in-life phase or at macroscopic evaluation post euthanasia.

Cytological analysis of the synovial fluids recovered from the implanted knees at termination indicated that, at 4 weeks, 2 of 4 fluids from knees implanted with pSFTs had moderate cellularity and 1 of 4 fluids from knees implanted with an oSFT had marked cellularity. This was also the case for the synovial fluid from one of the knees implanted with a pSFT at 26 weeks which showed marked cellularity, however the presence of PMNs in these fluids indicated that the presence of infection could not be excluded. Importantly, for the knees implanted with pSFT, the levels of inflammation in the synovial fluids at 12 and 26 weeks was considered to be within normal limits and only adverse in the 26-week pSFT fluid which may have been infected. Histopathological evaluation of the tendon tissue and bone tunnels, revealed an inflammatory reaction comprising lymphocytes, PMNs, macrophages and plasma cells to both the decellularised pSFT and oSFT allografts at 4 weeks which reduced by 26 weeks. Overall, while the pSFT elicited a slightly higher inflammatory reaction than the oSFT control, this did not impair graft performance in terms of biological integration and anchorage. Microscopic evidence of acceptable local tissue effects were observed with both graft types, especially from 12 weeks of implantation with evidence of advanced cellular ingrowth and ligamentisation in the intra-articular graft and calcification, ossification and formation of Sharpey's fibers at the graft/bone junctions. Inflammation is the initial response to tissue injury and hence the presence of inflammatory cells within the tissues at 4 weeks was not unexpected. It is well documented that ACL reconstruction with autografts and allografts in both humans [68,69] and sheep [66,[70], [71], [72]] involves an initial or inflammatory phase followed by tissue remodelling and maturation in the longer term. It is evident that an initial inflammatory response is necessary for subsequent integration and constructive remodelling of ACL grafts.

In order to gain greater insight into the nature of the host response within the intra-articular grafts, antibodies to markers for lymphocytes (CD3 for T-cells; CD19 for B-cells) and macrophages were employed. Antibodies to MAC 387 detect migration inhibitory factor related protein (MRP14 [48]), expressed in mononuclear phagocytes and polymorphonuclear leukocytes [49]. MRP14 identifies recently infiltrating monocytes/macrophages [50]. Antibodies to CD163 bind to the high affinity scavenger receptor for the haemoglobin-haptoglobin complex and in humans, CD163 is considered a marker for M2 macrophages [[40], [41], [42],^73^]. In sheep CD163 is expressed by cells in a similar localisation as CD11b + macrophages in lymph nodes [44]. Here, CD163 was utilised as a marker for tissue macrophages, putatively M2-type macrophages. Antibodies to CD80 bind to the co-stimulatory molecule B7.1 which is expressed by M1 macrophages [43,[45], [46], [47]].

The lymphocytes present in the explanted decellularised pSFT and oSFT allograft tissues were identified as CD3^+^ T-cells. Semi-quantitative scoring for lymphocytes and CD3^+^ cells was similar. Proliferating cells (Ki-67+) were apparent within the inflammatory infiltrates at 4 weeks, perhaps indicating active proliferation of CD3^+^ cells, however proliferating cells were rare at 12 and 26 weeks. There was a notable absence of any CD19^+^ cells or CD80^+^ cells in any of the tissues and MAC 387+ cells were only detected in significant numbers in the 4-week explants. There was no evidence to suggest an ongoing CD3^+^ T-cell mediated immune rejection response in the form ligament degradation, necrosis or activated CD80^+^ M1-type macrophages in the decellularised pSFT or oSFT grafts. It is possible that the grafts incited a Th2 –restricted response as has been reported for SIS in mice [74], although further investigation would be required to test this hypothesis. However, in support of this, the host response to the grafted tissues did appear to become dominated by CD163+ M2-macrophages. In the 4-week explants, CD163+ cells demarcated the decellularised and cellularised tissue regions appearing to be the “pioneer” cells infiltrating the matrix of the decellularised pSFT grafts. CD163+ cell numbers increased from 4 to 12-weeks and then began to decline by 26-weeks to levels similar to those in the native ovine ACL tissues. Interestingly, M2-macrophages (ED-2+; rat equivalent of CD163) have also been identified to have an anabolic pro-regenerative role in a rat model of ACL reconstruction with a syngeneic tendon graft [75].

A high proportion of the stromal cells in the native ovine ACL tissues expressed α-SMA, indicating myofibroblasts [51]. A high proportion of the cells growing into the grafted tissues at 4 weeks expressed α-SMA and at 12 and 26-weeks the density of α-SMA + cells in the explanted decellularised pSFT and oSFT tissues was similar to native ovine ACL. Myofibroblasts play an important role in the maintenance of matrix homeostasis of ligamentous tissues and matrix contraction and restructuring during healing [52], suggesting that these cells may have played a role in the ligamentisation observed from 12 weeks. Population of the implanted tissues by progenitor cells was investigated using CD271 (nerve growth factor receptor) and CD34. CD271 is a marker for multipotential mesenchymal stromal cells [53]. CD34, conventionally considered to be a marker for haematopoietic progenitors is now recognised as a general marker for progenitor cells [56,57]. In the native ovine ACL and explanted tissues the vascular endothelium was strongly CD34^+^ and antibodies to CD34 also weakly stained stromal cells, potentially indicating progenitor cells. CD34^+^ was a useful marker for neo-vascularisation of the explanted tissues, which was evident at the tissue margins at 4 weeks, greatest at 12-weeks and reduced at 26 weeks. CD271+ cells were present in low numbers specifically in perivascular locations in the native ovine ACL, presumably identifying pericytes. CD 271+ cells were identified in the same location in all explanted tissues, being most abundant at 12-weeks and reducing at 26-weeks. CTGF is a matricellular protein involved in the regulation of cellular activity and ECM production as well as fibrosis [54]. Moderate levels of CTGF were expressed by cells in the native ovine ACL, perhaps indicating that intrinsic CTGF expression is a feature of a dynamically loaded tissue, since cyclic strain has been shown to increase CTGF expression in ACL fibroblasts [55]. CTGF was expressed by cells populating the grafted tissues, at similar levels to those in native ovine ACL at 26-weeks.

Cellular population of the grafted tissues was accompanied by matrix expression of tenascin-C. Tenascin-C is an extracellular matrix glycoprotein important in regulating cellular differentiation and proliferation. In normal adult tendons, tenascin-C is expressed in regions transmitting high levels of mechanical force and during wound healing of tendon grafts [60,61]. Collagen Type III makes up circa 10% of the collagen in the ACL and its early expression in tendon tissues used for ACL reconstruction has been interpreted as a sign of ligamentisation [58]. Collagen III expression precedes collagen I expression during wound healing of ligaments [59]. Collagen III expression was associated with cellular repopulation in the explanted tissues, increasing over the 26 week period, indicating matrix remodelling associated with the histological evidence of ligamentisation.

Overall, by 26 weeks the cellular population of the explanted tissues was similar to native ACL with evidence that matrix remodelling was ongoing. At 4 and 12 weeks, recruitment of a constructive cell infiltrate appeared to be orchestrated by CD163+ M2 macrophages. Studies conducted by the Badylak group have characterized the macrophage response to a range of biological scaffolds *in vivo* and identified CD 163+ M2 macrophages as promoting constructive tissue remodelling [13,46,76]. This is the first study of a decellularised xenogeneic tissue for ACL replacement in a functional large animal model that has attempted to characterise the tissue remodelling process and supports the emerging paradigm for the role of M2 macrophages in constructive tissue remodelling of appropriately decellularised biological scaffolds. Interestingly, the oSFT allograft appeared to undergo similar cellular events despite containing non-viable cellular components. The reasons for this are not entirely clear, however it is well documented that in the sheep model, ACL allografts are remodelled over time with no evidence of overt immune rejection. Thus, it might be postulated that the fresh-frozen ovine tendon allografts contain the necessary cues and cell-guidance micro-structure to modulate the host response, despite containing residual cell remnants.

After 26 weeks implantation, the biomechanical properties of the oSFT allograft and decellularised pSFT grafts were comparable. An increase in mechanical parameters compared with those implanted in contralateral cadaveric knees (pSFT(C)) indicated integration of the grafts over time. A change in failure mode was also observed, moving from failure at the graft interface in the pSFT(C) grafts to ligamentous failure by week 26. Importantly, no grafts tested after 26 weeks failed at the graft-tunnel interface. This suggests that the remodelling observed in histological analysis was accompanied by improvements in graft stability. Although not the primary purpose of this study, grafts at 26 weeks were also compared with the biomechanical properties of the cadaveric contralateral ACLs. Neither type of graft had restored mechanical function to the level of the native ovine ACL, at 26 weeks, the failure load of the decellularised pSFT was 45% and oSFT allograft was 52% of the native ovine ACL (1114 ± 225 N). The linear stiffness of the decellularised pSFT was 46% and oSFT allograft was 43% of the native ovine ACL (162 ± 52 N mm^−1^). The data compared favourably to other studies reported in the literature at similar time points [27,29,33,34]. For example, Hunt et al. [34] reported a failure load of 313.8 N (20% of native ovine ACL) and a linear stiffness of 58.6 N.mm-1 (40% of native ovine ACL) for superficial flexor digitorum tendon allograft in sheep at 24 weeks. Mayr et al. [36] recorded higher values for linear stiffness (146.0 ± 69.3 N. mm^−1^) and failure load (807.9 ± 344.7 N) for flexor digitorum superficialis tendon allografts in sheep at 24 weeks, but this was 56% and 46% of the values recorded for linear stiffness and failure load of the native ovine ACL in their study. It is not clear at what time point the biomechanical properties of the native ACL are achieved, if ever, by an allograft in sheep [36]. Since the current study was limited to 26-weeks, it is not known whether there would be any difference in the longer term biomechanical performance of the decellularised pSFT compared to the oSFT allograft.

This study had several limitations. Since the study was conducted in sheep, the data cannot be directly translated to humans. The study was limited to 26 weeks, and although the time course for mechanical stability of ACL grafts in sheep is unclear, it is probable that this can take over a year [34,36]. The number of replicates analysed histologically and immunohistochemically at each time point was small (n = 4) and hence the study lacked statistical power. For this reason, quantitative histomorphometric analysis was not conducted. Longer term studies with increased numbers of animal replicates were not justified when this study was designed since, at the time, there were no published studies of functional ACL replacement in a large animal model with a decellularised xenogeneic biological scaffold and the outcome was uncertain.

## 5 Conclusions

This study provides evidence for the initial integration and functional performance of an “off the shelf” decellularised xenograft scaffolds as an alternative to current allograft replacement of the ACL. The study also provides new insight into the host response to tendon allografts and functional biological scaffolds used for ACL reconstruction. The study supports the emerging paradigm for a role of M2 macrophages in constructive tissue remodelling of appropriately decellularised biological scaffolds.

## CRediT author statement

**Jennifer Helen Edwards**: Formal analysis, Investigation, Data curation, Writing – original draft, Writing – Review & editing, Visualisation, Project administration. **Gemma Louise Jones**: Formal analysis, Investigation, Writing – Review & editing, Project administration. **Anthony Herbert**: Methodology, Formal analysis, Investigation, Data curation, Writing – Review & editing. **John Fisher**: Conceptualization, Methodology, Resources, Writing – Review & editing, Supervision, Project administration, Funding acquisition. **Eileen Ingham**: Conceptualization, Methodology, Formal analysis, Investigation, Resources, Writing – Review & editing, Supervision, Project administration, Funding acquisition.

## Declaration of competing interest

The authors declare the following financial interests/personal relationships which may be considered as potential competing interests: E. Ingham and J. Fisher are shareholders in Tissue Regenix Group Plc.

## Data Availability

The raw data required to reproduce these findings are available to download from https://doi.org/10.5518/898. The processed data required to reproduce these findings are available to download from https://doi.org/10.5518/898.
